# Cancer Anxiety Mediates the Association Between Satisfaction With Medical Communication and Psychological Quality of Life After Prophylactic Bilateral Salpingo-Oophorectomy

**DOI:** 10.3389/fpsyg.2022.840931

**Published:** 2022-03-09

**Authors:** Cristina Zarbo, Agostino Brugnera, Luigi Frigerio, Chiara Celi, Angelo Compare, Valentina Dessì, Rosalba Giordano, Chiara Malandrino, Federica Paola Sina, Maria Grazia Strepparava, Isadora Vaglio Tessitore, Mariangela Ventura, Robert Fruscio

**Affiliations:** ^1^Unit of Epidemiological and Evaluation Psychiatry, IRCCS Istituto Centro San Giovanni di Dio Fatebenefratelli, Brescia, Italy; ^2^Department of Human and Social Sciences, University of Bergamo, Bergamo, Italy; ^3^Department of Obstetrics & Gynaecology, Hospital Papa Giovanni XXIII, Bergamo, Italy; ^4^Clinical Psychology Unit, ASST-Monza, Monza, Italy; ^5^Gynaecologic Surgery Unit, ASST-Monza, Monza, Italy; ^6^Department of Medicine and Surgery, University of Milan-Bicocca, Milan, Italy; ^7^Private Practitioner, Bergamo, Italy

**Keywords:** prophylactic bilateral salpingo-oophorectomy, quality of life, communication, BRCA, ovarian cancer

## Abstract

**Background:**

Prophylactic Bilateral Salpingo-Oophorectomy (PBSO) reduces the risk of developing ovarian cancer. However, the psychological mechanisms that may affect post-surgery Quality of Life (QoL) among patients who underwent PBSO are still largely unknown. Thus, this study aimed at exploring the direct and indirect associations of satisfaction with medical communication and cancer anxiety on post-surgery QoL among women at high risk of developing ovarian cancer.

**Method:**

Fifty-nine women (mean age: 50.64 ± 6.7 years) who underwent PBSO took part in this cross-sectional study, filling out a sociodemographic and clinical questionnaire, a battery of validated psychological measures and an *ad hoc* developed scale for the assessment of cancer anxiety. We first examined the correlations among all variables of interest, and then tested if cancer anxiety mediated the association between satisfaction with medical communication and post-surgery psychological QoL, controlling both for time from surgery and education.

**Results:**

Post-surgery psychological QoL was unrelated from any sociodemographic or clinical variable. Cancer anxiety had a significant direct negative effect on psychological QoL, while satisfaction with medical communication had a significant positive direct effect on it. Finally, cancer anxiety significantly mediated the association between satisfaction with medical communication and post-surgery psychological QoL.

**Discussion:**

Results suggest that post-surgery psychological QoL of patients who underwent PBSO may be increased with interventions, delivered in a genetic counselling setting, targeting quality of medical communication and cancer anxiety.

## Introduction

Each year, ovarian cancer accounts for an estimated 239,000 new cases and 152,000 deaths worldwide (Ferlay et al., 2015). Despite recent improvements in detection and treatment, mortality is still high and data relating to its distribution are still alarming. In this context, genetic tests that evaluate the inherited tendency to develop ovarian cancer are particularly useful, as they allow to prevent cancer’s onset and, consequently, to reduce the mortality rates (Bleiker et al., 2003). In the gynecological field, recent studies have shown that women with the BRCA (BReast CAncer) gene 1 or 2 mutation have a significantly increased lifetime risk of developing both breast (from 39 to 85%) and/or ovarian cancer (from 10 to 63%)(Ford et al., 1998; Meijers-Heijboer et al., 2001; Antoniou et al., 2003). In case of a family history of breast\gynecological cancer or of a BRCA 1/2 mutation, the medical team may recommend (based on age, the desire for parenting, and other clinical considerations for that specific patient) an intensive screening or even perform a prophylactic surgery. In some cases, surgery (i.e., prophylactic mastectomy and/or prophylactic bilateral salpingo-oophorectomy) is recommended as it substantially reduces the risk of developing breast or ovarian cancer (Schrag et al., 1997; Meijers-Heijboer et al., 2001).

### Quality of Life After PBSO

Despite the significant psychological impact that performing a Prophylactic Bilateral Salpingo-Oophorectomy (PBSO) may represent, few studies have examined the patients’ post-surgery Quality of Life (QoL), reporting mixed results. That is, women who underwent PBSO report a reduction of both worry related to the risk of developing cancer and psychological distress after the surgery (i.e., a higher positive wellbeing and a reduction in anxious or depressive symptoms), but at the same time they often show menopausal symptoms, an alteration of body image (including feeling less physically attractive), a reduced sexual functioning (i.e., satisfaction with sexual functioning, libido reduction, vaginal dryness, reduced sexual arousal, and lubrification) and vitality, poorer physical and social functioning, physical role limitations, and higher pain intensity (Meiser et al., 2000; Elit et al., 2001; Tiller et al., 2002; van Oostrom et al., 2003; Aziz et al., 2005; Madalinska et al., 2005; Bresser et al., 2007a; Fang et al., 2009; Michelsen et al., 2009; Razzaboni et al., 2012; Finch et al., 2013; Borreani et al., 2014; D’Alonzo et al., 2018; Hickey et al., 2021a; Islam et al., 2021; Kershaw et al., 2021; Philp et al., 2021). However, in most cases, the negative effects of PBSO (i.e., vaginal discomfort and impairment of quality of life) significantly decrease after 6 months or 1 year after surgery, even if a smaller percentage of patients experience long-term impairments, such as cancer-related distress (from 18% to 22/27%) and anxiety (19%), which need to be further investigated (Bresser et al., 2007a; Fang et al., 2009; Finch et al., 2013). Interestingly, Hickey et al. (2021b) found an increase in depressive and anxious symptoms after a PBSO surgery. During the 12-months post-surgery follow-up depressive symptoms persisted while anxiety symptoms returned to baseline (Hickey et al., 2021b).

Literature has identified several risk and protective factors for long-term post-surgery QoL outcomes among women who undergo BPSO. For example, being a BRCA mutation carrier, having younger children, the loss of a relative due to breast/ovarian cancer, limited open communication about the test results within the family, changes in relationships with relatives, being in premenopausal status at operation, doubting about the validity of the test results, a greater risk perception, higher educational level, and occupying an executive position are important predictors of lower post-surgery general QoL (Fry et al., 2001; van Oostrom et al., 2003; Bresser et al., 2007b; Touboul et al., 2011; Tucker et al., 2016). Conversely, positive coping strategies like having comforting thoughts are predictive of higher post-surgery general QoL (Bresser et al., 2007b). In addition, anxiety and cancer anxiety (i.e., anxiety symptomatology specifically related to the worry of developing cancer) may also play a significant role in affecting post-surgery QoL.

### Medical Communication and Post-surgery Outcomes

One could argue that satisfaction with medical communication about the genetic counselling, procedures related to the surgery, and post-surgery recovery may constitute an important protective factor for a good post-surgery psychological QoL and recovery. It is well known that an efficient and good communication—characterized by a positive patient-physician alliance, information sharing, and by a greater ability to foster patient’s feelings of control and mastery—has an overall positive effect on the psychological wellbeing of patients (Hack et al., 2005). In particular, a good medical communication influences the perception of control about the treatment, the overall compliance with the treatment, frequency of symptoms, self-management skills, engagement in preventive health behaviors, satisfaction for the surgery’s choice, QoL, anxiety, and depression (Kretsch et al., 2014). To date, literature has highlighted that women at high risk for cancer frequently claim for insufficient information to make an informed decision about the choice to carry out the surgery (Hallowell, 1998). The study of Trister et al. (2021) found that 43% of the women undergoing BSO did not feel well informed about what to expect post-operatively.

They further report the need for supportive communications (Babb et al., 2002), in particular relative to the effects of surgical menopause, the link between Hormone Replacement Therapy (HRT) and breast cancer (Meiser et al., 2000), and potential physical and emotional effects of the surgery (Hallowell, 1998).

### Literature Limitations and Current Study

Literature on this population has several shortcomings. In accordance with Finch and Narod (2011), well performed follow-up studies on the long-term health and quality of life consequences of BPSO are still missing. Furthermore, to date, very few studies focused on the role of medical communication in the field of genetic counselling or prophylactic surgery, and no studies focused on cancer anxiety after BPSO, nor on the relationship between anxiety and QoL in this population.

In order to overcome these limits, we tested a mediation model in a population of PBSO patients, where the association between satisfaction with medical communication (i.e., the level of satisfaction regarding the medical communication received during the surgery pathway) and post-surgery psychological QoL is mediated by cancer anxiety. In accordance with Stark and House (2000), we hypothesized that a greater satisfaction with medical communication will reduce the overall levels of cancer anxiety, leading to a greater post-surgery psychological QoL.

## Materials and Methods

### Participants and Procedure

A total of 59 consecutive women (mean age: 50.64 ± 6.7 years) who underwent PBSO volunteered for this retrospective study. Participants were recruited from January to July 2019 in two hospitals located in North Italy and were approached during their yearly follow-up visit after the PBSO surgery (median time from surgery: 2 years). We included women aged 18+ years, who underwent PBSO in the last 5 years for different reasons (i.e., history of breast cancer, BRCA 1/2 genetic mutation, presence of ovarian diseases, or a family history of cancer), free of past or concurrent neurological and psychiatric diseases or severe medical conditions, and proficient in written and spoken Italian. Sociodemographic and clinical data, including means and standard deviations for all psychological variables, have been reported in Table 1. The majority of the participants attended high school (54.2%), was married (74.6%), and was currently working (72.9%). From a clinical point of view, they were mostly diagnosed with a BRCA 1/2 genetic mutation (81.6%) or had a history of breast cancer (67.8%) treated with a mastectomy (66.1%).

**Table 1:** Frequencies, percentages, means, and standard deviations for all sociodemographic, clinical, and psychological variables examined in our sample of women who underwent Prophylactic Bilateral Salpingo-Oophorectomy (PBSO; *N* = 59).

Variable	
Age: *mean* (*SD*)	50.64(6.7)
Education: *n* (%)	
*Middle Schools*	18(30.5)
*High Schools*	32(54.2)
*Bachelor’s Degree*	1(1.7)
*Master’s Degree*	8(13.6)
Marital Status: *n* (%)	
*Engaged*	2(3.4)
*Married*	44(74.6)
*Widow*	1(1.7)
*Single*	7(11.9)
*Separated*	3(5.1)
*Divorced*	2(3.4)
Work status: *n* (%)	
*Student*	1(1.7)
*Employed full-time*	19(32.2)
*Employed part-time*	19(32.2)
*Self-employed*	5(8.5)
*Unemployed*	11(18.6)
*Retired*	4(6.8)
Having Children: *n* (%)	48(81.4)
**Clinical information**	
Having a Chronic medical condition: *n* (%)	5(8.5)
Having BRCA 1/2 genetic mutation: *n* (%)	48(81.6)
History of breast cancer: *n* (%)	40(67.8)
Previous mastectomy: *n* (%)	
*Yes, for treating breast cancer*	33(66.1)
*Yes, for preventing breast cancer*	3(5.1)
History of chemotherapy: *n* (%)	29(49.2)
History of radiotherapy: *n* (%)	28(47.5)
Age at surgery: *mean (SD)*	48.8(6.8)
Menopause status before surgery: *n* (%)	34(57.6)
Years since surgery: *mean (SD)*	1.83(1.54)
**Psychological variables:** *mean (SD)*	
*Physical QoL*	15.2(2.31)
*Psychological QoL*	14.4(2.39)
*Social QoL*	14.8(2.61)
*Satisfaction with medical communication*	8.05(2.07)
*Body image distress*	8.92(8.15)
*Sexual functioning*	51.3(20.9)
*Sexual distress*	11.2(10.7)
*Cancer anxiety*	8.37(6.49)

### Measures

#### Primary Measures

##### Satisfaction With Medical Communication

Satisfaction with medical communication received in relation to the surgery was assessed through a single item on a Likert-type scale (i.e., from 0 = “*completely unsatisfied*” to 10 = “*completely satisfied*”). The item asked patients *“From 0 (completely unsatisfied) to 10 (completely satisfied), how much satisfied are you with the medical information you received before and after the prophylactic surgery?”*

##### Quality of Life

Post-surgery QoL (physical, psychological, and social; α ranges in the current study: 0.71 to 0.84) was assessed using the Italian version of the World Health Organization Quality of Life -bref (WHOQoL-bref; De Girolamo et al., 2000). The WHOQoL-bref is a self-administered questionnaire comprising 26 questions on the individual’s perceptions of their health and wellbeing over the previous 15 days. Responses are on a 5-point Likert-type scale (from 1 = “*disagree*”\"*not at all*” to 5 = “*completely agree*”\"*extremely*”). The scale covers four domains, namely Physical health (seven items), Psychological QoL (six items), Social relationships (three items), and Environment QoL (eight items). There are also two items that are examined separately: question 1 asks about an individual’s overall perception of quality of life and question 2 asks about an individual’s overall perception of their health. In the present study, the Environment subscale was not included in the protocol. The total scores of each subscale range from 4 to 20, with higher scores indicating a greater QoL in that specific domain. Each score is calculated through a detailed syntax described in the manual of the instrument (World Health Organization, 1996).

##### Cancer Anxiety

Cancer anxiety was examined through an *ad hoc* developed cancer anxiety scale, composed by two Likert-type items investigating both “*Cancer rumination*” and “*Cancer risk perception*.” The items asked patients to rate from 0 (“*not at all*”) to 10 (“*very much*”) how much they experienced the following mental states: (a) “*I have recurring thoughts about cancer*” (Cancer rumination); “*I have the perception that my life is constantly threatened”* (Cancer risk perception). Cancer anxiety total score is calculated by the mean of the two items and ranges from 0 to 10. Higher scores indicate higher cancer anxiety.

#### Secondary Measures

Sociodemographic and clinical information were collected using a structured *ad-hoc* self-report questionnaire.

##### Sexual Functioning

Sexual functioning was assessed through the Italian version of the Female Sexual Function Index (FSFI; α in the current study: 0.96; Filocamo et al., 2014). The FSFI is a 19-items scale aimed at targeting the six domains of female sexual functioning: desire (two items, range 2–10), arousal (four items, range 0–20), lubrication (four items, range 0–20), orgasm (three items, range 0–15), satisfaction (three items, range 2–15), and pain (three items, range 0–15). Items of all subscales, except for desire and satisfaction, are rated on a 6-point Likert-type scale (from 0 to 5), while the items of the subscales desire and satisfaction are rated on a 5-point Likert-type scale (from 1 to 5). Items 14, 15, and 16 are reverse-scored. The total score ranges from 4 to 95, with higher scores indicating a better sexual functioning.

##### Sexual Distress

Sexual distress was investigated through the Female Sexual Distress—revised (FSDS-r; α in the current sample: 0.97; Derogatis et al., 2008), a 13-items scale aimed at assessing distress related to sexuality. Respondents indicate how often each of the listed sexual-related problems (e.g., absence of desire and feeling of guilty) has caused distress in the previous 7 days on a Liker-type scale from 0 (“*never*”) to 4 (“*always*”). Total score ranges from 0 to 52, with higher scores indicating greater sexual distress. The Italian translated version provided by the authors (copyright: American Foundation for Urological Disease Inc.) has been used in this research study.

##### Body Image Distress

Body image distress was assessed through the Italian version of the Body Image Scale (BIS; α in the current sample: 0.93; Cheli et al., 2016). The BIS is a 10-item measure of different dimensions of body image in cancer patients. It uses a 4-point response scale (from 0 = “*not at all*,” to 3 = “*very much*”). Total scores range from 0 to 30, with higher scores indicating worse symptoms and distress or more body image concerns. In this study, we aimed at assessing body image beliefs in the last week and in relation to surgery consequences.

### Statistical Analysis

We performed an *a priori* power analysis (*F* test family and linear multiple regression with two predictors as statistical test) using the software G*POWER 3.1. We first extracted effect sizes from a similar study on QoL of women at high risk for breast cancer who underwent a prophylactic surgery (f2 = 0.17; Brandberg et al., 2008). The *a priori* power analysis evidenced that 60 participants were required to detect a medium-high effect size (*f*^2^ = 0.17) on psychological QoL (*α* = 0.05; Power = 0.80). Hence, our study had adequate power.

Data were initially screened for assumptions. Thus, we tested for the presence of univariate and multivariate outliers, and examined the univariate normality of all variables of interest (Tabachnick and Fidell, 2007). We then investigated the possible relationships between sociodemographic, clinical, and psychological variables, and post-surgical psychological QoL, through Pearson’s or Spearman’s *r* correlation coefficients.

**Figure 1:**
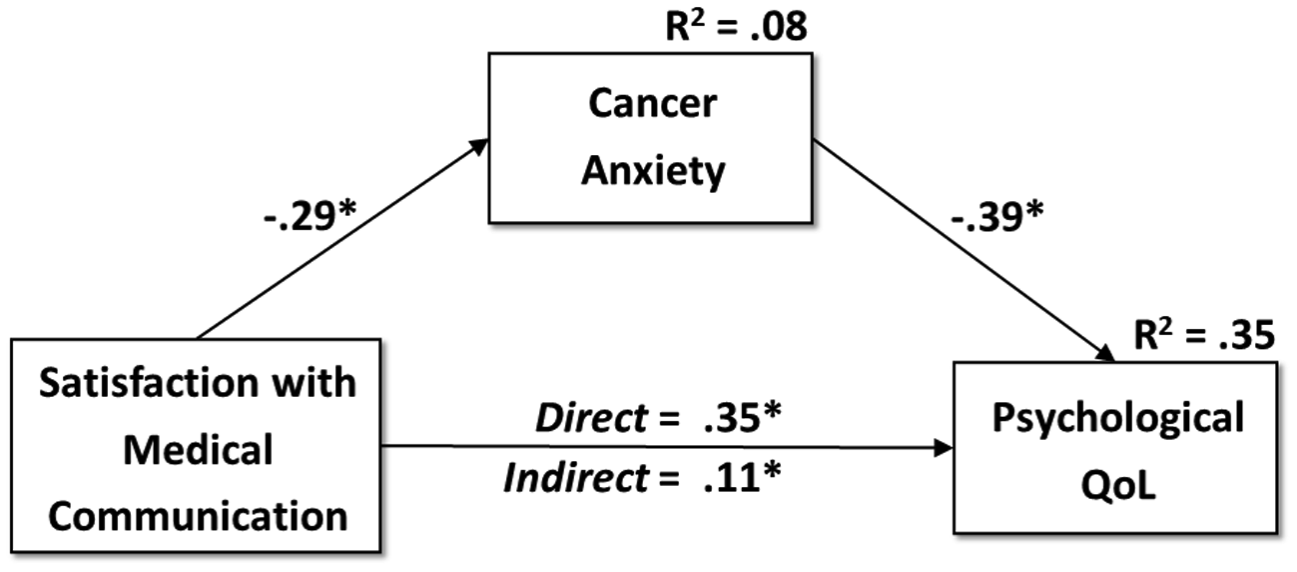
The mediation model in the sample of patients who underwent Prophylactic Bilateral Salpingo-Oophorectomy (PBSO; *N* = 59). ^*^p ≤ 0.05.

We tested our main hypothesis on the mediating effect of cancer anxiety on the association between satisfaction with medical communication (i.e., the independent variable) and post-surgery psychological QoL (i.e., the dependent variable), while controlling both for time from surgery and educational level (grouped into “low education” and “high education,” with a cut-off of 8 years of education), through a bootstrapped test of mediation analysis. The analysis was conducted by using Hayes’ PROCESS Mediation Model version 3.4, which is a conditional process modeling program that utilizes an ordinary least squares-based path analytical framework to test for both indirect and direct effects (Hayes, 2017). Our hypothesis was tested adopting Model 4 in PROCESS (i.e., a simple mediator model; see Figure 1). We used random sampling with replacement to generate 5,000 bootstrap samples. This approach produces bias-corrected estimates of the standard errors of parameter estimates and a bias-corrected 95% confidence interval (CI) of the mediation effects. If the 95% CI for the hypothesized indirect effect does not include zero, the indirect effect is significant at *p* ≤ 0.05 (Hayes, 2017). Effect sizes were reported and interpreted according to guidelines (Cohen, 1988).

All analyses were run with SPSS version 26 (SPSS, 2019). All tests were two tailed, and a value of *p* ≤ 0.05 was considered significant.

## Results

### Preliminary Analyses

Preliminary analyses evidenced that all variables were normally distributed, while no univariate or multivariate outliers were identified. Further, the cancer anxiety scale specifically developed for this study evidenced good psychometric properties: a Principal Component Analysis with one extracted component explained 93.65% of the variance, and both items had a saturation of 0.97 with the factor “cancer anxiety.” The internal consistency was also good, with a Cronbach’s α of 0.93.

Correlational analyses (see Table 2) suggested positive associations between post-surgery psychological QoL and social QoL (*r* = 0.631; *p* < 0.001), physical QoL (*r* = 0.724; *p* < 0.001), and satisfaction with medical communication (*r* = 0.365; *p* < 0.001), with medium-to-large effects. In addition, post-surgery psychological QoL was negatively correlated with body image distress (*r* = −0.406; *p* < 0.001) and cancer anxiety (*r* = −0.490; *p* < 0.001), with medium effects. Finally, post-surgery psychological QoL was unrelated from sociodemographic and clinical variables, nor from the time passed since prophylactic surgery (see Table 2).

**Table 2:** Zero-order correlations between post-surgery psychological Quality of Life (QoL) and all variables of interest (*N* = 59).

Variable(s)	*r* with Psychological QoL	Sample Size *r*
Age	0.039	59
Education (dichotomized as “low” and “high”)	0.156	59
Socioeconomic Status	−0.042	47
Familiar status (dichotomized as “not in a relationship” and “in a relationship”)	−0.047	59
Working status (dichotomized as “not working” and “working”)	−0.068	59
Motherhood	−0.050	59
BRCA 1/2 genetic mutation	0.173	59
Time from surgery	−0.186	59
Menopause status before surgery	0.024	59
Previous radiotherapy	−0.136	59
Previous chemotherapy	0.050	58
Previous mastectomy	−0.178	59
Previous breast cancer	−0.121	59
Social QoL	0.631^**^	59
Physical QoL	0.724^**^	59
Sexual distress	0.034	49
Sexual functioning	0.009	46
Satisfaction with medical communication	0.365^**^	59
Body image distress	−0.406^**^	59
Cancer anxiety	−0.490^**^	59

*BRCA 1/2 (BReast CAncer gene 1 or 2)*.

** *p ≤ 0.001.*

### Mediation Model

We tested our main hypothesis on the mediating effect of cancer anxiety (i.e., the mediator) on the association between satisfaction with medical communication (i.e., the independent variable) and post-surgery psychological QoL (i.e., the dependent variable), while controlling both for time from surgery and educational level. Our mediation model (see Figure 1) evidenced that the satisfaction with medical communication was a significant negative direct predictor of cancer anxiety (*β* = −0.285, *p* = 0.032), with medium effects. Further, cancer anxiety had a significant negative direct effect on post-surgical psychological QoL (*β* = −0.392, *p* = 0.001), while satisfaction with medical communication was a significant positive predictor of the dependent variable (*β* = 0.345, *p* = 0.004), with medium effects. Finally, cancer anxiety mediated the association between satisfaction with medical communication and post-surgical psychological QoL (*β* = 0.112, SE = 0.058, 95% CI [0.023, 0.245]), with medium effects This model accounted for approximately 35% of the variance in the dependent variable.

## Discussion

This study sought to investigate if cancer anxiety mediated the association between satisfaction with medical communication and post-surgery psychological QoL, in a sample of women who underwent PBSO. Findings suggested that, among patients who underwent PBSO, a greater satisfaction with medical communication decreases cancer anxiety, which in turn improves post-surgical psychological QoL. To the best of our knowledge, very few studies have investigated QoL after BPSO, and even fewer examined the predictors of long-term post-surgery QoL within this sample. Our study provided a significant contribution to this emerging topic, focusing on the needs and shortcomings of the current literature.

We found that post-surgery psychological QoL was unrelated from any sociodemographic and clinical (i.e., BPSO or health related) factors. That is, the psychological QoL of women who underwent BPSO seems to be modulated by other individual factors, such as the psychological ones. Our results differ from those reported by Touboul et al. (2011) where the post-surgical QoL of women who underwent BPSO was positively associated with higher education and an executive working position. We also found that menopause status before surgery, BRCA 1/2 genetic mutation diagnosis, history of breast cancer, and of previous cancer treatments were not associated with post-surgery psychological QoL. These results are similar to most of those reported both in Finch et al. (2013) and Touboul et al. (2011), even if Finch et al. (2013) found that a history of breast cancer predicted a lower physical QoL after BPSO.

Furthermore, we found that body image distress was negatively associated with post-surgery psychological QoL. Therefore, post-surgery psychological QoL may be partially explained by the perceived body image resulted from the surgery (i.e., distress for weight gain and scars). This finding is particularly interesting and need further investigation since most of the current literature has focused exclusively on body image distress after a mastectomy (Aygin and Cengiz, 2018; Huang and Chagpar, 2018).

Counselling and quality of communication in this field are important. Trister et al. (2021) highlighted that, despite patients usually receive pre-surgery counselling, there is a need to provide supplemental patient materials in preparing patients for what to expect after surgery. We provided more information about the significant role of counselling in this context. Indeed, we found that satisfaction with medical communication significantly affect both post-surgery cancer anxiety and psychological QoL. In particular, our result suggested that a greater satisfaction with medical communication directly increases post-surgery psychological QoL and reduces cancer anxiety. In turn, reduced cancer anxiety leads to a greater psychological QoL. In accordance with Kretsch et al. (2014), we hypothesize that a greater quality of medical communication, focused on providing accurate information on (i) the risk condition or genetic predisposition, (ii) possible prophylactic surgeries, and (iii) post-surgery recovery, using an emphatic and patient-centered style, may be associated with a greater satisfaction with medical communication. This, in turn, may enhance both the patients’ self-efficacy competencies and their focus on an internal locus of control (i.e., the beliefs that the outcomes of one own’s actions are results of one own abilities and choices). Thus, thanks to a greater satisfaction with medical communication, patients who underwent PBSO may experience psychological changes that promote psychological QoL and reduce perceived risk and rumination on cancer.

## Limitations

Despite the clinical significance of our findings, the limited sample size, the retrospective assessment of the satisfaction with medical communication, and the use of an *ad-hoc* measure of cancer anxiety may limit the generalizability of our findings. The retrospective assessment of the satisfaction with medical information could have been affected by a recall bias, even if we tried to partially control for this systematic error entering time from surgery as a covariate in our mediation model. In addition, satisfaction with medical communication and quality of medical communication are partially overlapping—but still distinct—dimensions. Indeed, the former may be influenced by other personal or clinical factors (e.g., personal expectations, complications related to the surgery, previous surgery for breast cancer, symptomatology perception, and the impact of induced menopause), not *per se* reflecting the quality of the communication. Finally, satisfaction with medical communication may also be affected by health literacy and education, even if we tried to partially control for this including educational level as covariate in the mediation model.

## Conclusion

Findings support the importance to guarantee high quality of counselling provided to women undergoing PBSO. To reach this goal, it could be helpful (i) to include mental health practitioners within the genetic screening procedure or as part of the pre-surgical evaluation team and (ii) to deliver specific communication-based trainings for the medical team aimed at reducing the cancer anxiety experienced by patients. High-quality medical communication and greater patients’ satisfaction could help those who undergo PBSO to better cope with the long-term psychological *sequelae* of prophylactic surgery, reducing the possible distress, and increasing—in the long run—their overall sense of wellbeing and QoL. Better satisfaction with BPSO and subsequent greater QoL might be in turn related to a wider diffusion of prophylactic surgical treatments and the maximization of ovarian cancer prevention.

## Data Availability Statement

The raw data supporting the conclusions of this article will be made available by the authors, without undue reservation.

## Ethics Statement

The studies involving human participants were reviewed and approved by the Hospital Papa Giovanni XXIII, Bergamo. The patients/participants provided their written informed consent to participate in this study.

## Author Contributions

CZ, AB, LF, VD, and RF: conceptualization, methodology, data curation, data analysis, original draft, and writing—review and editing. CC, AC, RG, CM, FS, MS, IT, and MV: conceptualization, methodology, and writing—review and editing. All authors contributed to the article and approved the submitted version.

## Conflict of Interest

The authors declare that the research was conducted in the absence of any commercial or financial relationships that could be construed as a potential conflict of interest.

## Publisher’s Note

All claims expressed in this article are solely those of the authors and do not necessarily represent those of their affiliated organizations, or those of the publisher, the editors and the reviewers. Any product that may be evaluated in this article, or claim that may be made by its manufacturer, is not guaranteed or endorsed by the publisher.

## References

[ref2] Antoniou A. Pharoah P. D. Narod S. Risch H. A. Eyfjord J. E. Hopper J. L.. (2003). Average risks of breast and ovarian cancer associated with BRCA1 or BRCA2 mutations detected in case series unselected for family history: a combined analysis of 22 studies. Am. J. Hum. Genet. 72, 1117–1130. doi: 10.1086/375033, PMID: 12677558 PMC1180265

[ref3] Aygin D. Cengiz H. (2018). Life quality of patients who underwent breast reconstruction after prophylactic mastectomy: systematic review. J. Breast Cancer 25, 497–505. doi: 10.1007/s12282-018-0862-8, PMID: 29721811

[ref4] Aziz A. Bergquist C. Nordholm L. Möller A. Silfverstolpe G. (2005). Prophylactic oophorectomy at elective hysterectomy: effects on psychological well-being at 1-year follow-up and its correlations to sexuality. Maturitas 51, 349–357. doi: 10.1016/j.maturitas.2004.08.018, PMID: 16039407

[ref5] Babb S. A. Swisher E. M. Heller H. N. Whelan A. J. Mutch D. G. Herzog T. J.. (2002). Qualitative evaluation of medical information processing needs of 60 women choosing ovarian cancer surveillance or prophylactic oophorectomy. J. Genet. Couns. 11, 81–96. doi: 10.1023/A:1014571420844 26141655

[ref6] Bleiker E. M. Hahn D. E. Aaronson N. K. (2003). Psychosocial issues in cancer genetics--current status and future directions. Acta Oncol. 42, 276–286. doi: 10.1080/02841860310004391 12899498

[ref7] Borreani C. Manoukian S. Bianchi E. Brunelli C. Peissel B. Caruso A.. (2014). The psychological impact of breast and ovarian cancer preventive options in BRCA1 and BRCA2 mutation carriers. Clin. Genet. 85, 7–15. doi: 10.1111/cge.12298, PMID: 24117034

[ref8] Brandberg Y. Sandelin K. Erikson S. Jurell G. R. Liljegren A. Lindblom A.. (2008). Psychological reactions, quality of life, and body image after bilateral prophylactic mastectomy in women at high risk for breast cancer: a prospective 1-year follow-up study. J. Clin. Oncol. 26, 3943–3949. doi: 10.1200/JCO.2007.13.9568, PMID: 18711183

[ref9] Bresser P. Seynaeve C. Van Gool A. Niermeijer M. Duivenvoorden H. van Dooren S.. (2007a). The course of distress in women at increased risk of breast and ovarian cancer due to an (identified) genetic susceptibility who opt for prophylactic mastectomy and/or salpingo-oophorectomy. Eur. J. Cancer 43, 95–103. doi: 10.1016/j.ejca.2006.09.009 17095208

[ref10] Bresser P. Van Gool A. Seynaeve C. Duivenvoorden H. Niermeijer M. Van Geel A.. (2007b). Who is prone to high levels of distress after prophylactic mastectomy and/or salpingo-ovariectomy? Ann. Oncol. 18, 1641–1645. doi: 10.1093/annonc/mdm274, PMID: 17660493

[ref11] Cheli S. Agostini A. Herd-Smith A. Caligiani L. Martella F. Fioretto L. (2016). The Italian version of body image scale reliability and sensitivity in a sample of breast cancer patients. Psicoterapia Cognitiva e Comportamentale 22:42000. doi: 10.1037/t56642-000

[ref12] Cohen J. (1988). Statistical Power Analysis for the Behavioral Sciences. 2nd Edn. New York, NY: Academic Press.

[ref13] D’Alonzo M. Piva E. Pecchio S. Liberale V. Modaffari P. Ponzone R.. (2018). Satisfaction and impact on quality of life of clinical and instrumental surveillance and prophylactic surgery in BRCA-mutation carriers. Clin. Breast Cancer 18, e1361–e1366. doi: 10.1016/j.clbc.2018.07.015, PMID: 30122348

[ref14] De Girolamo G. Rucci P. Scocco P. Becchi A. Coppa F. D’Addario A.. (2000). Quality of life assessment: validation of the Italian version of the WHOQOL-brief. Epidemiol. Psichiatr. Soc. 9, 45–55. doi: 10.1017/s1121189x00007740, PMID: 10859875

[ref15] Derogatis L. Clayton A. Lewis-D'Agostino D. Wunderlich G. Fu Y. (2008). Validation of the female sexual distress scale-revised for assessing distress in women with hypoactive sexual desire disorder. J. Sex. Med. 5, 357–364. doi: 10.1111/j.1743-6109.2007.00672.x, PMID: 18042215

[ref16] Elit L. Esplen M. Butler K. Narod S. (2001). Quality of life and psychosexual adjustment after prophylactic oophorectomy for a family history of ovarian cancer. Familial Cancer 1, 149–156. doi: 10.1023/a:1021119405814, PMID: 14574171

[ref17] Fang C. Y. Cherry C. Devarajan K. Li T. Malick J. Daly M. B. (2009). A prospective study of quality of life among women undergoing risk-reducing salpingo-oophorectomy versus gynecologic screening for ovarian cancer. Gynecol. Oncol. 112, 594–600. doi: 10.1016/j.ygyno.2008.11.039, PMID: 19141360 PMC2697574

[ref18] Ferlay J. Soerjomataram I. Ervik M. Dikshit R. Eser S. Mathers C.. (2015). "GLOBOCAN 2012, Cancer Incidence and Mortality Worldwide: IARC CancerBase No. 11". (Lyon, France: International Agency for Research on Cancer).

[ref19] Filocamo M. T. Serati M. Li Marzi V. Costantini E. Milanesi M. Pietropaolo A.. (2014). The female sexual function index (FSFI): linguistic validation of the Italian version. J. Sex. Med. 11, 447–453. doi: 10.1111/jsm.12389, PMID: 24224761

[ref20] Finch A. Metcalfe K. A. Chiang J. Elit L. McLaughlin J. Springate C.. (2013). The impact of prophylactic salpingo-oophorectomy on quality of life and psychological distress in women with a BRCA mutation. Psychooncology 22, 212–219. doi: 10.1002/pon.2041, PMID: 21913283

[ref21] Finch A. Narod S. A. (2011). Quality of life and health status after prophylactic salpingo-oophorectomy in women who carry a BRCA mutation: a review. Maturitas 70, 261–265. doi: 10.1016/j.maturitas.2011.08.001, PMID: 21893388

[ref22] Ford D. Easton D. Stratton M. Narod S. Goldgar D. Devilee P.. (1998). Genetic heterogeneity and penetrance analysis of the BRCA1 and BRCA2 genes in breast cancer families. Am. J. Hum. Genet. 62, 676–689. doi: 10.1086/301749, PMID: 9497246 PMC1376944

[ref23] Fry A. Busby-Earle C. Rush R. Cull A. (2001). Prophylactic oophorectomy versus screening: psychosocial outcomes in women at increased risk of ovarian cancer. Psycho-Oncology 10, 231–241. doi: 10.1002/pon.512, PMID: 11351375

[ref24] Hack T. F. Degner L. F. Parker P. A. (2005). The communication goals and needs of cancer patients: a review. Psychooncology 14, 831–845. doi: 10.1002/pon.949 16200519

[ref26] Hallowell N. (1998). You don’t want to lose your ovaries because you think “I might become a man”. Women's perceptions of prophylactic surgery as a cancer risk management option. Psycho-Oncology 7, 263–275. doi: 10.1002/(SICI)1099-1611(199805/06)7:3<263::AID-PON307>3.0.CO;2-Q 9638787

[ref27] Hayes A. F. (2017). Introduction to Mediation, Moderation, and Conditional Process Analysis: A Regression-Based Approach. New York: Guilford publications.

[ref28] Hickey I. Jha S. Wyld L. (2021a). The psychosexual effects of risk-reducing bilateral salpingo-oophorectomy in female BRCA1/2 mutation carriers: A systematic review of qualitative studies. Gynecol. Oncol. 160, 763–770. doi: 10.1016/j.ygyno.2020.12.001, PMID: 33317909

[ref29] Hickey M. Moss K. M. Brand A. Wrede C. D. Domchek S. M. Meiser B.. (2021b). What happens after menopause?(WHAM). Gynecol Oncol 161, 527–534. doi: 10.1016/j.ygyno.2021.02.001, PMID: 33583580

[ref30] Huang J. Chagpar A. B. (2018). Quality of life and body image as a function of time from mastectomy. Ann. Surg. Oncol. 25, 3044–3051. doi: 10.1245/s10434-018-6606-3, PMID: 29947006

[ref31] Islam R. M. Davis S. R. Bell R. J. Tejada-Berges T. Wrede C. D. Domchek S. M.. (2021). A prospective controlled study of sexual function and sexually related personal distress up to 12 months after premenopausal risk-reducing bilateral salpingo-oophorectomy. Menopause 28, 28, 748, 748–755, 755. doi: 10.1097/GME.0000000000001766, PMID: 33739311

[ref32] Kershaw V. Wyld L. Jha S. J. E. J. O. O. (2021). The impact of risk reducing bilateral salpingo-oophorectomy on sexual function in BRCA1/2 mutation carriers and women with lynch syndrome: A systematic review and meta-analysis. Eur. J. Obstet. Gynecol. Reprod. Biol. 265, 7–17. doi: 10.1016/j.ejogrb.2021.08.001, PMID: 34416580

[ref33] Kretsch M. Chone L. Spitz E. (2014). Illness perceptions and quality of life in cancer: does communication with the physician matter? Proc. Am. Soc. Clin. Oncol. 32:e20576. doi: 10.1200/jco.2014.32.15_suppl.e20576

[ref34] Madalinska J. B. Hollenstein J. Bleiker E. Beurden M. V. Valdimarsdottir H. B. Massuger L.. (2005). Quality-of-life effects of prophylactic salpingo-oophorectomy versus gynecologic screening among women at increased risk of hereditary ovarian cancer. J. Clin. Oncol. 23, 6890–6898. doi: 10.1200/JCO.2005.02.626, PMID: 16129845

[ref35] Meijers-Heijboer H. van Geel B. van Putten W. L. Henzen-Logmans S. C. Seynaeve C. Menke-Pluymers M. B.. (2001). Breast cancer after prophylactic bilateral mastectomy in women with a BRCA1 or BRCA2 mutation. N. Engl. J. Med. 345, 159–164. doi: 10.1056/NEJM200107193450301 11463009

[ref36] Meiser B. Tiller K. Gleeson M. A. Andrews L. Robertson G. Tucker K. M. (2000). Psychological impact of prophylactic oophorectomy in women at increased risk for ovarian cancer. Psycho-Oncology 9, 496–503. doi: 10.1002/1099-1611(200011/12)9:6<496::aid-pon487>3.0.co;2-z, PMID: 11180584

[ref37] Michelsen T. M. Dørum A. Dahl A. A. (2009). A controlled study of mental distress and somatic complaints after risk-reducing salpingo-oophorectomy in women at risk for hereditary breast ovarian cancer. Gynecol. Oncol. 113, 128–133. doi: 10.1016/j.ygyno.2008.12.024, PMID: 19178933

[ref38] Philp L. Alimena S. Ferris W. Saini A. Bregar A. Del Carmen M.. (2021). Patient reported outcomes after risk-reducing surgery in patients at increased risk of ovarian cancer. Gynecol. Oncol. 164, 421–427. doi: 10.1016/j.ygyno.2021.12.017 34953629

[ref39] Razzaboni E. Tazzioli G. Andreotti A. De Matteis E. Cortesi L. Federico M. (2012). Prophylactic surgery to reduce the risk of developing breast cancer: issues and clinical implications. Current Bioact. Comp. 8, 94–103. doi: 10.2174/157340412799079237

[ref41] Schrag D. Kuntz K. M. Garber J. E. Weeks J. C. (1997). Decision analysis—effects of prophylactic mastectomy and oophorectomy on life expectancy among women with BRCA1 or BRCA2 mutations. N. Engl. J. Med. 336, 1465–1471. doi: 10.1056/NEJM199705153362022, PMID: 9148160

[ref01] SPSS I. (2019). IBM SPSS software version 26. IBM Press.

[ref42] Stark D. House A. (2000). Anxiety in cancer patients. Br. J. Cancer 83, 1261–1267. doi: 10.1054/bjoc.2000.1405, PMID: 11044347 PMC2408796

[ref43] Tabachnick B. G. Fidell L. S. (2007). Using Multivariate Statistics. Boston, MA: Pearson Education, Inc.

[ref44] Tiller K. Meiser B. Butow P. Clifton M. Thewes B. Friedlander M.. (2002). Psychological impact of prophylactic oophorectomy in women at increased risk of developing ovarian cancer: a prospective study. Gynecol. Oncol. 86, 212–219. doi: 10.1006/gyno.2002.6737, PMID: 12144830

[ref45] Touboul C. Uzan C. Ichanté J. L. Caron O. Dunant A. Dauchy S.. (2011). Factors associated with altered long-term well-being after prophylactic salpingo-oophorectomy among women at increased hereditary risk for breast and ovarian cancer. Oncologist 16, 1250–1257. doi: 10.1634/theoncologist.2010-0336, PMID: 21765195 PMC3228172

[ref46] Trister R. Jacobson M. Nguyen P. Sobel M. Allen L. Narod S. A.. (2021). Patient reported experiences following laparoscopic prophylactic bilateral salpingo-oophorectomy or salpingectomy in an ambulatory care hospital. Fam. Cancer 20, 103–110. doi: 10.1007/s10689-020-00208-y, PMID: 32964297

[ref47] Tucker P. E. Bulsara M. K. Salfinger S. G. Tan J. J.-S. Green H. Cohen P. A. (2016). The effects of pre-operative menopausal status and hormone replacement therapy (HRT) on sexuality and quality of life after risk-reducing salpingo-oophorectomy. Maturitas 85, 42–48. doi: 10.1016/j.maturitas.2015.12.004, PMID: 26857878

[ref48] van Oostrom I. Meijers-Heijboer H. Lodder L. N. Duivenvoorden H. J. van Gool A. R. Seynaeve C.. (2003). Long-term psychological impact of carrying a BRCA1/2 mutation and prophylactic surgery: a 5-year follow-up study. J. Clin. Oncol. 21, 3867–3874. doi: 10.1200/jco.2003.10.100, PMID: 14551306

[ref49] World Health Organization (1996). “WHOQOL-BREF: introduction, administration, scoring and generic version of the assessment: field trial version, December 1996”. World Health Organization.

